# Clinical Aspects of Dermatitis Associated with *Dirofilaria repens* in Pets: A Review of 100 Canine and 31 Feline Cases (1990–2010) and a Report of a New Clinic Case Imported from Italy to Dubai

**DOI:** 10.1155/2011/578385

**Published:** 2011-12-13

**Authors:** Walter Tarello

**Affiliations:** Department of Small Animal Internal Medicine, Pet Connection Veterinary Clinic, P.O. Box 450288, Dubai, United Arab Emirates

## Abstract

Cutaneous dirofilariasis is a parasitic disease caused by the mosquito-borne filarial nematodes *Dirofilaria (Nochtiella) repens*, living in the subcutaneous tissue of dogs, cats, wild carnivores, and humans. Cases have been recently reported also from Germany, Czech Republic, Hungary, Ukraine, Russia, Austria, Switzerland, France, The Netherlands, and the Middle East. *D. repens* is not widely known to cause chronic pruritic dermatitis in animals. Dermatological signs observed in 100 canine clinic cases were pruritus (100%), erythema (79%), papulae (62%), focal or multifocal alopecia (55%), hyperkeratosis (18%), crusting (14%), nodules (12%), acantosis (5%), and eczema (3%). Signs other than dermatological were conjunctivitis (46%), anorexia (35%), vomiting (26%), fever (25%), lethargy (20%), and lymph-adenomegaly (10%). A case imported from Italy to Dubai is described. The opportunistic role of *D. repens* might explain the presence of asymptomatic carriers, the concurrent observation of nondermatological signs, and the development of dermatitis in a subgroup of parasitized dogs.

## 1. Introduction

Two main filarial parasites affect domestic carnivorous in Europe: *Dirofilaria immitis*, a parasite of the cardiovascular system, and *Dirofilaria *(*Nochtiella*)* repens*, a parasite of the subcutaneous connective tissue of dogs, cats, wild carnivores, and humans [1]. Aside these, pets can be less frequently infected by *Acanthocheilonema* (syn*. Dipetalonema*) *reconditum* and *Cercopithifilaria* (syn. *Acanthocheilonema*) *grassii *[2].

**Figure 1:**
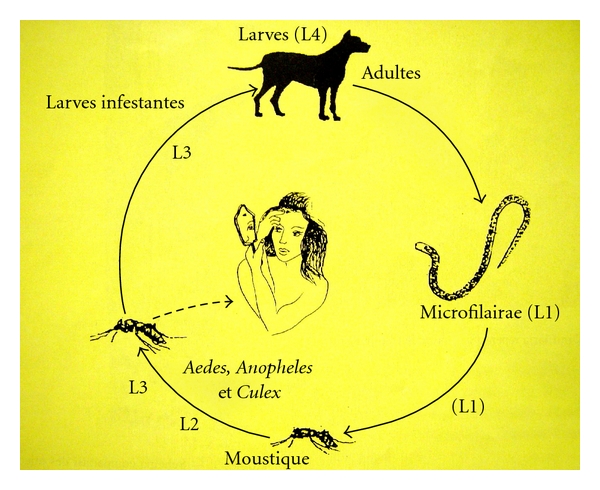
*Dirofilaria repens* cycle.

Subcutaneous dirofilariasis due to *Dirofilaria repens *is endemic in Southern and Eastern Europe, and many parts of Africa and Asia [1]. Dogs, cats, and wild carnivores are final hosts of *D. repens *and constitute the only source of accidental infestation for humans, in the presence of a competent population of mosquito vectors, including the Asian tiger mosquito *Aedes albopictus *and *Culex pipiens *[3]. Human cases have been recorded mainly in Italy, France, Spain, and Sri Lanka [3]. During the recent years, animal and human infection with *D. repens *has been detected in new areas of the world, including the Alps [4], Ukraine [5], the Middle East [6–8], and Germany [9]. Strict quarantine regulations seldom prevent propagation of *D. repens*, because the infection becomes patent only after 6–10 months and the adult parasite can live 2–4 years in the subcutaneous tissues of dogs [10, 11]. In infected cats [12–14] and dogs [15–17], diagnosis is based upon the presence of pruritic skin lesions, the finding of *D. repens* microfilariae, and a negative test for circulating *D. immitis* antigens [16]. The combined use of concentration techniques (Knott) and heartworn antigen tests improves the accuracy to 98% [18]. Although the parasitosis may appear asymptomatic [10, 15], a seasonal variance exists in the number of circulating microfilariae, with peaks in August-September, associated with cyclic clinical manifestations, such as pruritus, erythema, and alopecia, caused by mechanical, toxic and immunomediated actions of the parasite [16]. In a control group of microfilaraemic asymptomatic dogs, 43% developed pruritic skin lesions within 5 months [16]. Experimentally infected dogs show microfilaraemia 6 months after inoculation even in the presence of only one *D. repens *male [9]. Mosquitoes suck the blood of infected dogs, ingesting microfilariae (larvae L1) which develop into L2 and infective L3 larvae within 10–20 days (Figure 1). During a mosquito's blood meal L3 larvae penetrate into the subcutaneous tissues of a dog, where they molt to L4 larvae and remain for 6-7 months, before developing into adults (Figure 1) [10]. Males parasites measure 5–7 cm and females 10–17 cm in length.

**Figure 2:**
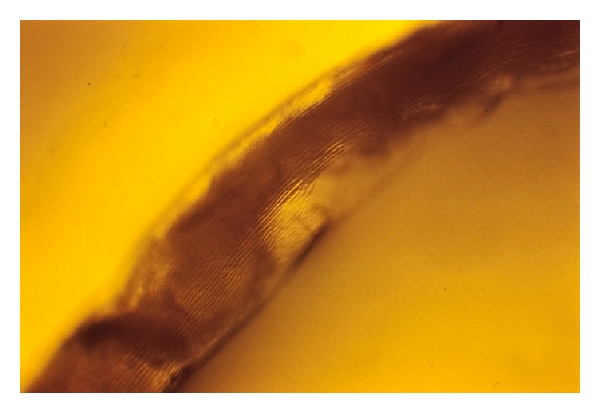
Longitudinal ridges are evident in a *Dirofilaria repens* adult nematode.

**Figure 3:**
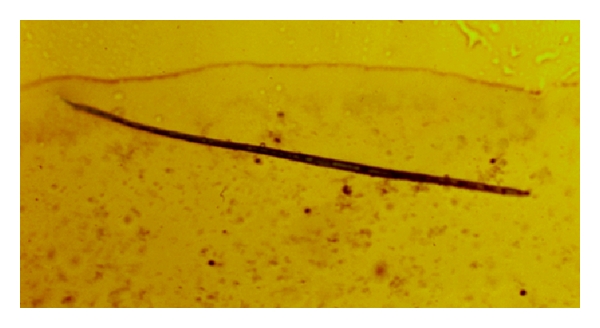
Microfilaria of *Dirofilaria repens* (×200).

**Figure 4:**
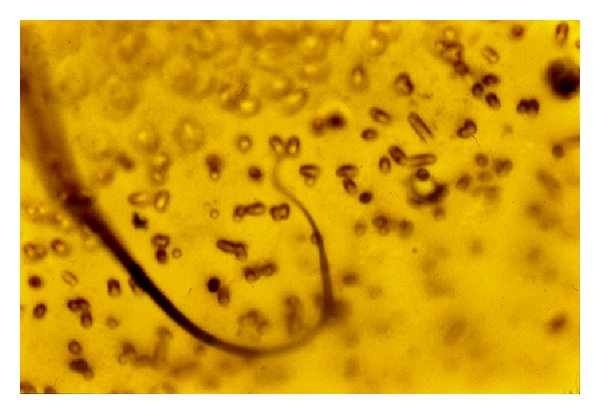
Tail of Acanthocheilonema (Dipetalonema) reconditum microfilaria (×400).

Cuticular longitudinal ridges constitute the main difference with *D. immitis* [11] (Figure 2). Unsheathed *D. repens* microfilariae measure 325–375 microns in length and 7–8.3 microns in width (Figure 3), showing a cephalic space roundish and empty as well as a tail larger than those of *D. immitis *and* Acanthocheilonema *(*Dipetalonema*) *reconditum* (Figure 4) [10].

**Figure 5:**
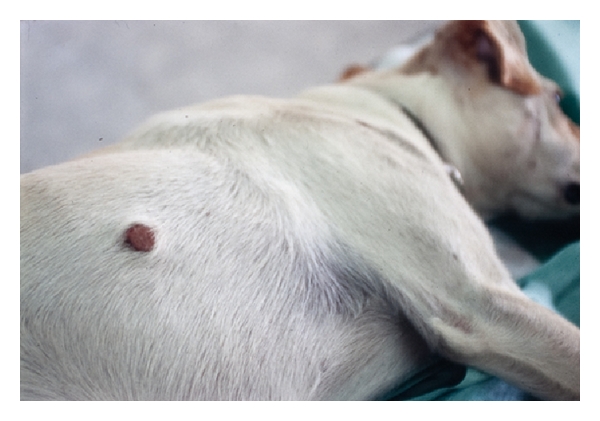
Nodule on the flank of a dog from Italy (Alessandria province) containing an adult female *Dirofilaria repens* parasite.

 Due to their location, adult nematodes are rarely found, occasionally being recovered from skin nodules (Figure 5) [17].

**Figure 6:**
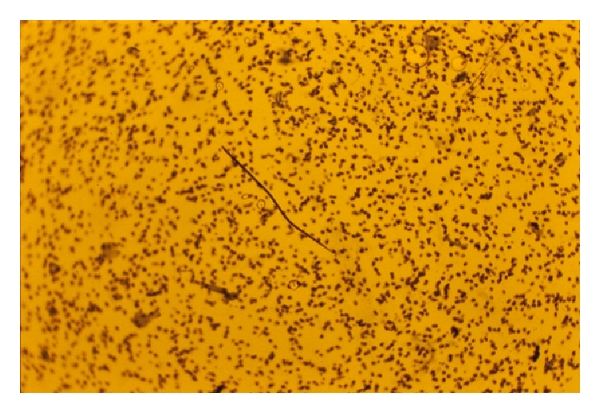
Microfilaria of *D. repens*.

Nevertheless, detection of species-specific microfilariae is diagnostic for *Dirofilaria repens *infections (Figure 6) [10–17].

*Dirofilaria immitis* lives in the heart and large vessels (heartworm) of dogs and is occasionally reported in abscess-like lesions in the skin, especially on the legs [19]. These locations are erratic and unusual. Pruritic papulonodular dermatitis has been associated with these locations and probably is the result of hypersensitivity to the presence of adults in the skin. Dogs with heartworm-associated dermatitis typically show chronic itching, ulcerated papules, nodules, and plaques. Lesions are most commonly found on the head and on the limbs but can be anywhere [19].

A third species, *Acanthocheilonema (Dipetalonema) reconditum*, affects dogs from Europe, America, and Asia with no evidence of clinical signs. However, Hargis and colleagues reported a filariasis, apparently due to an *anthocheilonema-*like parasite, in 10 dogs from the western Unites States showing pruritic papules and plaques with alopecia, scarring, erythema, ulceration, and crusting [20]. The head, neck, and shoulders were most commonly affected. Three consecutive ivermectin injections cleared the infection [20]. *Acanthocheilonema reconditum *microfilariae are 4-5 microns in width only, much thinner than those of *immitis* or *repens,* and their tail is frequently hook-shaped (Figure 4). 

A fourth very rare dermatitis-causing filarial parasite, *Cercopithifilaria* (syn. *Acanthocheilonema*) *grassii *is transmitted by ticks and found mainly in Central Italy [2].

However, the most important agent of subcutaneous dirofilariasis in dogs and humans remains *Dirofilaria (Nochtiella) repens *[1, 10, 16].

## 2. Vectors

A number of *Anopheles, Aedes, and Culex *mosquito species are its vectors, including the Asian Tiger mosquito *Aedes albopictus *[21]*, Aedes caspius, Aedes vexans, Anopheles maculipennis, Culex modestus, *and* Culex pipiens *[10, 16].

## 3. Diagnosis

Diagnosis is based upon the presence of pruritic skin lesions, the finding of *D. repens* microfilariae, and a negative test for circulating *D. immitis* antigens [16]. Differentiation is also possible using the phosphatasic acid histochemical technique: the sediment of centrifuged blood is stained with alfa naphthyl ASTR phosphate which evidences two areas of phosphatasic acid activity in brick red colour for *D. immitis *microfilariae and one area only, at the posterior end, for *D. repens *[10]. PCR is today available as well. Differential diagnosis includes atopic dermatitis and other pruritics ecto-parasitosis [16].

## 4. Epidemiology

Endemic areas of canine and feline subcutaneous dirofilariasis have been described in the Mediterranean countries where human cases have been reported [3]. This is in accord with the notion that geographic distribution of human dirofilariasis follows the distribution of animal dirofilariasis [8]. Dogs, cats, and wild carnivores, in fact, are final hosts of *D. repens *and constitute the only source of accidental infection to humans, in the presence of a competent population of mosquito vectors [1].

Cats are apparently less susceptible than dogs. Italy is the only European country where *repens *microfilariae have been found in cats, even though the first feline case I diagnosed in Italy actually originated from Camargue (France) [4]. In 2000, I examined 11 autochthonous feline cases in the area between Pavia, Alessandria, and Casale Monferrato in Piedmont, northern Italy [12, 13], which is considered the most endemic area in the world [1, 3].

Nineteen further feline cases (bringing the total to 31) were diagnosed in my practice in Central Italy: 14 from Umbria (Trasimeno Lake), 2 from Tuscany (Chiusi lake), and 3 from Marche (Fermo), all associated with pruritic dermatitis, including alopecia, erythema, papulae, crusting, and lichenification [14]. These findings were recently confirmed by the isolation of 5 new *repens *feline cases from Central Italy, using Knott's modified method, serology for *Dirofilaria immitis* antigen, and PCR [21].

 Adult nematodes, 1 male and 1 female, have been recovered only in one cat from Kiev, Ukraine [5]. Microfilaraemia is commonly seen in cats from Southeast Asia [10]. Dog constitute the main reservoir and main definitive host for *Dirofilaria repens*, with the highest prevalences being found in dogs from Sri Lanka (60%), Iran (61%), and Italy (30%, Po River Valley) [16].

A national survey carried out in France (1986) revealed that 1.3% of 5,502 dogs were parasitized by *Dirofilaria repens *[10]. French Army dogs living in endemic areas in Southern France had 22% prevalence rate and 50% of these animals had mixed infection with* immitis *[10]. Presence of *repens *microfilariae is common in dogs from Greece (22%) and Spain (9%) as well [10, 16]. Microfilaraemia has been observed in dogs from all regions of Italy, mostly in Piedmont, Tuscany, and Sicily [22].

It is interesting to note that in the last years in Italy the endemic area of *Dirofilaria repens *has considerably expanded [21, 22].

Mixed infection with *Dirofilaria immitis *has been seen in 12% of dogs affected by *Dirofilaria repens *in endemic areas of Italy where both parasites are present. Suitable climates and presence of vectors can, therefore, facilitate the diffusion of this filarial worm [3–17].

The highest prevalence in reported in Serbia, with 49.2% of dogs found positive to *D. repens *in a recent survey [23], confirming 28 human cases recorded in the last 40 years in the same Country [24].

To effectively prevent mosquito-borne diseases, owners and veterinarians should be aware of the risks associated with the geographic movements of pets.

Questioning the owner regarding history of travel and living areas of pets has become essential in order to obtain a correct diagnosis and effective therapy.

To stress the importance of collecting all anamnestic data to build up a good *case history*, I often recall that my first encounter with *Dirofilaria repens* happened in a nonendemic area, Aosta Valley (North-Western Italy), near the border with France and Switzerland. It was the case of a cat with a 3-year history of itching dermatitis previously residing for 2 years in Camargue (south France) [4]. The location, Camargue, and the presence of a cutaneous syndrome unresponsive to previous therapies led to a search that culminated in my first diagnosis of *Dirofilaria repens *[4]. 

## 5. History

It is probably not a coincidence that the earliest documented report of subcutaneous dirofilariasis comes from southern France, and dates back more than 400 years.

In 1566, Amatus Lusitanus (1511–1568), a Portuguese physician, reported the first clinic case of ocular filariasis in a 3-year-old child in southern France that most probably can be attributed to *Dirofilaria repens *[1]. His report suggests that similar cases were not uncommon in southern France at that time.

Three centuries later, Italian ophthalmologist Addario (1885) removed a worm from the eyelid of a woman in Milan [1]. The worm was named *Filaria conjunctivae *because of its location in the eye. Later on, when worms where submitted to identification, this name was dropped in favour of the current denomination, *Dirofilaria repens, *which is now recognized as a cause of subcutaneous, subconjunctival, and pulmonary nodules [3]. Itching, swelling, and tenderness of the affected site (arm, eyelid, chin, temporal area, or testicle) are common in human subcutaneous dirofilariasis [3]. Italy is the country most affected, recording more than 200 cases, followed by Sri Lanka, France, Ukraine, Greece, and the Balkans [1, 3].

## 6. Veterinary Discovery

In veterinary medicine, *D. repens* was first described by Bonvicini in a dog from Bologna, Italy in 1910. The parasite was then speciated in France by Railliet and Henry (1911) [10].

It was not until 1953 that the nematode was isolated again in nine adult specimens by two clinicians, Guilhon and Graber, in the subcutaneous tissues of dogs living in the Paris area of France [10]. In 1954, Professor Giulio Ajmerito, who would become later my teacher of Pharmacology and Pharmacotherapy at the Veterinary School of Turin, first recognized *D. repens *as a cause of pruritic dermatitis in a dog from Piedmont, Italy [10]. In his Italian paper, a dog showing a chronic itching dermatitis was carrying *D. repens *microfilariae in the blood and was successfully treated with an arsenical medicament [10]. During the sixties, the parasite was isolated again by Restani and colleagues in six dogs from central Italy affected by pruritic dermatitis relapsed after medication with corticosteroids and antibiotics [10]. Dogs were successfully treated with an arsenic-based drug named Caparsolate, confirming that they were effectively infected by a filarial worm.

In 1987, Beaufils and Martin-Granel found a dog coinfected with *Hepatozoon canis, Leishmania, *and* Dirofilaria repens *in southern France [10].

Two other French authors, Cazelles and Montagner, observed in 1996 two dogs coinfected with *Leishmania donovani *and *Dirofilaria repens *[10]. 

These findings might be discharged as anecdotal; however, they are important in the light of recently cumulated evidence that *Dirofilaria repens *is an opportunistic parasite often manifesting clinical signs in association with concurrent immune-suppressive conditions such as babesiosis, erlichiosis, and leishmaniosis [16].

## 7. Pathogenicity

*D. repens *is not widely known to cause pruritic dermatitis, apparently persisting as a well-kept veterinary dermatological secret for at least one century. However, there is scientifically recognized and widely published evidence that common signs of the infection in pets are itch (pruritus), papulae, erythema, alopecia, crusting, hyperkeratosis, lichenification and acantosis [4–17]. Occasionally, subcutaneous nodules can be seen, made by a cyst enclosing an adult nematode [5, 10]. In most cases, however, no pathogenic signs are observed in animals carrying *repens* microfilariae [16]. As a consequence, detection of *D. repens *microfilariae in dogs is still regarded by many (vets) as irrelevant and not requiring treatment, although medical therapy would greatly decrease the risk of infection to humans and would help to eliminate cutaneous ailments in affected animals or to prevent their appearance or flaring.

The pathogenicity of the nematode, in fact, is still poorly understood, mainly because (a) skin lesions appear only in a subset of infected dogs and are not predictable; (b) the gastrointestinal signs and poor performance in symptomatic dogs are not strictly indicative of a filarial disease; (c) classic adulticide and microfilaricide treatments seldom produce complete clinical recovery and parasitological eradication [16].

### 7.1. Review of Clinical Signs Observed in 100 Dogs (1990–2010)

Dogs living in rural areas or with access to outdoor environments are usually more affected since the risk of mosquitoes bite is higher [16]. During summer and autumn, the larger number of circulating microfilariae microscopically observed in the blood increases the chance of cutaneous manifestations [10]. Skin symptoms tend to recur seasonally in spring to autumn during the second-third year and to become persistent after the fourth year of infection [16]. Pathogenic effects, either seasonal or permanent, are due to the cumulative action of increased number of microfilariae, increased number of adults, their autoimmune and toxic effects, and reinfection [16].

Affected dogs show first pruritus, manifested by localised scratching, licking, and biting. The itch initially mild will become progressively severe, causing self-traumatic lesions [10].

Dermatological signs observed in 100 canine cases of subcutaneous dirofilariasis examined between 1990 and 2010 were as follows: pruritus (100%), erythema (79%), papulae (62%), focal or multifocal alopecia (55%), hyperkeratosis (18%), crusting (14%), nodules (12%), acantosis (5%), eczema (3%), pyoderma (3%), and oedema (1%) [4, 6–8, 10, 11, 15–17]. Generally, 85% of dogs had at least one lesion on the posterior part of the body (lumbosacral region, hind limbs, and perianal area).

In the same 100-dog group, symptoms and signs other than dermatological were as follows: conjunctivitis (46%), anorexia (35%), vomiting (26%), fever (25%), lethargy (20%), and lymphadenomegaly (10%) [4, 6–8, 10, 11, 15–17].

Such general signs are not caused by *D. repens*. In fact, a recent report from Greece shows that there is no significant difference in sport performances between hunting dogs carrying *repens *microfilariae and healthy non-microfilaraemic dogs [25]. So what causes the general signs?

These are caused by underlying concurrent factors/agents that help the manifestation, persistence, and severity of clinical signs associated with subcutaneous dirofilariasis [4–8]. On the other hand, *Wolbachia* bacteria help *Dirofilaria immitis *manifestation, persistence, and severity [26]. In my experience, eradication of underlying conditions followed by therapy with adulticide and microfilaricide drugs is essential to the elimination of clinical signs and disappearance of microfilaraemia [10–17]. Healing is confirmatory of the diagnosis [16]. Obviously, the recovery speed depends upon the duration of the disease, the age of the animal, and the severity of lesions [16].

Subcutaneous dirofilariasis should be included in the differential diagnosis of pruritic dermatitis and the exclusionary diagnosis of atopic dermatitis in pets living in endemic areas [16].

### 7.2. Concurrent Infections Observed in 100 Dogs (1990–2010)

Reviewing concurrent infections found in 100 dogs diagnosed with *D. repens,* babesiosis was the most common (95%), followed by granulocytic ehrlichiosis (40%), *Leishmania* (4%), *Hepatozoon canis *(2%), *Ehrlichia canis* (1%), and *Ehrlichia platys *(1%), [4, 6–8, 10, 11, 15–17]. It is acknowledged that *Babesia *and *Ehrlichia *species induce immune suppression favoring opportunistic infections [27].

*Babesia* and *Ehrlichia* infections show common signs of illness such as fever, lethargy, anorexia, and vomiting [28–30]. Interestingly, these are also the most prevalent collateral signs observed in dogs diagnosed with subcutaneous dirofilariasis: anorexia (35%), vomiting (26%), fever (25%), and lethargy (20%) [4, 6–8, 10, 11, 15–17], thus confirming the claim of coinfection.

A recent study from Germany seems to confirm these findings. Pingen and colleagues [31] observed that 12% of dogs imported from Hungary carried *D. repens *microfilariae, and 19% were infected with *Babesia canis, *11.6% with* Anaplasma phagocytophilum*, the agent of canine granulocytic ehrlichiosis, and 1.6% with *Ehrlichia canis.*

### 7.3. Review of Clinical Signs Seen in 31 Cats (1990–2010)

Among 31 cats with subcutaneous dirofilariasis [4, 5, 12–14], symptoms observed more often were pruritus (100%), alopecia (77.4%), erythema (74.2%), papulae (51.6%), and crusting (29%).

Symptoms and signs other than dermatological were as follows: anorexia (35.5%), lymphadenomegaly (32.3%), pale mucous membranes (29%), lethargy (16%), conjunctivitis (16%), pain (16%), and fever (10%) [4, 5, 12–14]. Concurrent infection with haemobartonellosis (*Mycoplasma haemofelis* infection), or Feline Infectious Anemia, which is transmitted by fleas or ticks, was recorded in 25 (80%) out of 31 cats examined and its therapy with doxycycline (10 mg/kg, for 20 days) greatly contributed to the clinical resolution [4, 5, 12–14]. Doxycycline is important in the therapy of dirofilariasis because it also eradicates the *Wolbachia* spp. bacteria symbiotic of adult worms, causing their sterilization and death [26].

### 7.4. Clinic Canine Case Imported from Italy to Dubai

A 2-year-old male Maremman-Abruzzese shepherd dog, named *Cerchio,* originating from the Abruzzo region of Italy, was imported in January 2011 to Dubai and examined on May 7th 2011, because of a 1-month history of itching dermatitis, poor appetite, vomiting, and fever. Cutaneous lesions were characterized by erythema and papulae on elbows, hocks, head, neck and abdomen, eczema, and alopecia on head, neck, thorax, flanks, and abdomen.

*Leishmania* and heartworm antigen tests resulted negative, whereas the Knott's modified test performed on 1 milliliter whole blood showed the presence of a high number of *repens* microfilariae.

A Wright-stained fresh blood smear revealed the concurrent occurrence of *Babesia gibsoni *and granulocytic *Ehrlichia*-like organisms within some neutrophils.

Tickborne pathogens were treated first with the antibabesial drug imidocarb dipropionate (1 mL/17 kg, once a week for 4 times) associated with doxycycline at a rickettsial dosage (10 mg/kg/day, os, for 21 days). This treatment resolved the systemic clinical signs, such as anorexia, vomiting, and fever together with partial improvement of the cutaneous lesions and itching. Treatment with melarsomine (2.5 mg/kg, im. twice at an interval of 24 h) began 2-3 days after completing the therapy for babesiosis and ehrlichiosis and this led to a further improvement of cutaneous lesions including pruritus.

Ten days later, a microfilaricide therapy with ivermectin (50 mcgr/kg, sc.) completed the therapy.

Full dermatological recovery was met at the end of the therapy. As recently reported, autochthonous foci of canine and feline infections by *D. repens *exist in the Abruzzo region of Central Italy [21]. This case imported to Dubai from Italy confirms how easily filarial parasites with an incubation period of 6–8 months can be introduced in new areas [16] where suitable climate and presence of competent vectors [6] would facilitate the spread of the nematode [3].

### 7.5. Factors Influencing Clinical Signs

In epidemiologic surveys, subcutaneous dirofilariasis appears nonsymptomatic in a large number of animals, defined as healthy carriers [16]. However, when the parasitosis is not eradicated, cutaneous manifestations may appear in a subset of patients, in a frame time ranging from few months to several years. For instance, in a personal follow-up case study (Alessandria, Italy) on a group of 9 untreated microfilaraemic and asymptomatic dogs, 4 (44%) out of 9 patients developed pruritic skin lesions within 5 months [16]. This means that with time increased chances are to observe dermatological manifestations.

Cutaneous signs are caused by (1) capillary embolization of microfilariae, (2) movement of adults in the subcutaneous tissues, (3) immunological-allergic reactions to parasitic stages L3-L5 and microfilariae, and (4) toxins released by the parasites [10, 16].

Development of allergic and autoimmune reactions affecting the skin is common in parasitic diseases, including heartworm, depending on the number of parasites, the duration of the infection, and the age and nutritional status of the animal [16]. Experimentally infected dogs show microfilaraemia 6–8 months after inoculation even in the presence of only 1 male [9].

Production of microfilariae continues for several months and lasts up to 3 years [1, 16]. Females can produce up to 5,000 microfilariae per day [10].

The intensity of “parturition” increases during spring and summer, with peaks in August and September, associated with cyclic manifestation of pruritus, erythema, and alopecia [10, 16]. That is why, may be also due to concurrent reinfection, subcutaneous dirofilariasis shows seasonal periodicity in the first 2-3 years [10]. Nocturnal periodicity is not marked for *Dirofilaria repens *since at noon there is only 20–40% reduction in circulating microfilariae [10].

As a consequence of this, blood for the search of microfilariae can be drawn from dogs under examination at any time during the day without risking false negative results [16]. The adult worms reside in the subcutaneous tissues, where they live for as long as 4 years and release microfilariae that circulate in the blood [17]. The combined action of adults and microfilariae prolonged over months and years, in association with triggering agents/factors causing transient or permanent immune-suppression, contributes to the manifestation of itching and dermatological signs. The opportunistic role of *Dirofilaria repens *might well explain the presence of asymptomatic carriers, the concurrent observation of nondermatological signs and the development of dermatitis only in a subset of parasitized dogs [4, 6–8, 10, 11, 15–17] and cats [4, 5, 12–14]. Comparatively, development and sexual differentiation of adult nematodes is facilitated in human patients affected by primary or secondary immunodeficiences [1, 3].

## 8. Implications and Conclusions

A frequently asked question is as follows: should we treat dogs and cats with nonsymptomatic microfilaraemia [4–8, 10–17]? The answer is *yes* if we are working in areas where suitable vectors exist and human cases have been reported [1, 3].

*D. repens* should be treated in all affected animals, independently from the presence of clinical signs by (1) eliminating all predisposing/triggering factors/agents detected, (2) administering melarsomine to eliminate adults, (3) and ivermectin or spot-on solutions of imidacloprid/moxidectin to eradicate microfilariae [16, 31]. All dogs aged 6 months or more living in endemic areas should be tested by Knott's test, and medically treated if positive for *repens* microfilariae before being submitted to preventive medication with moxidectin/imidacloprid [32]. The apparent opportunistic role of *D. repens* might well explain the presence of asymptomatic carriers, the concurrent observation of nondermatological signs, and the development of dermatitis in a subgroup of parasitized dogs and cats [4–8, 10–17].
